# Risk factors for gallbladder polyps observed through second-look abdominal sonography in patients with fatty liver disease

**DOI:** 10.1097/MD.0000000000029643

**Published:** 2022-07-29

**Authors:** Shu-Hsien Lin, Kun-Ta Wu, Yi-Chun Chiu, Chih-Chi Wang, King-Wah Chiu

**Affiliations:** a Division of Hepato-Gastroenterology, Department of Internal Medicine, Kaohsiung Chang Gung Memorial Hospital and Chang Gung University College of Medicine, Kaohsiung, Taiwan; b Division of General Surgery, Department of Surgery, E-Da Hospital, Kaohsiung, Taiwan; c Division of General Surgery, Department of Surgery, Kaohsiung Chang Gung Memorial Hospital, Kaohsiung, Taiwan; d Liver Transplantation Centre, Kaohsiung Chang Gung Memorial Hospital, Kaohsiung, Taiwan.

**Keywords:** fatty liver disease, gallbladder polyp, health examination, metabolic syndrome, second-look abdominal sonography

## Abstract

Multiple studies have provided varied results on the relationship between gallbladder polyps (GBPs), fatty liver disease (FLD), and metabolic factors.

The purpose of this study was to determine the possible risk factors related to GBP formation in Taiwanese population through the use of health examinations.

In this retrospective study, 1311 subjects who underwent abdominal sonography for health evaluations from September 2019 to August 2020 were randomly enrolled. Baseline characteristics of the study subjects were recorded. Risk factors related to GBP formation were analyzed. All participants’ series of abdominal sonography examinations in our hospital were also retrospectively reviewed to reveal the presence of GBPs through second-look sonography.

Among 1311 participants, 946 participants (72.2%) had clinically evident FLD, as documented using abdominal sonography; GBPs were found in 233 (24.6%) subjects with FLD. The incidence of FLD was significantly associated with the presence of GBP (*P* < .001; OR: 4.16, 95% CI: 10.67–35.55). However, the severity of FLD was not found to be associated with GBP (*P* = .052). In a multi-variate analysis for risk factors for GBP formation, FLD (*P* < .0001, OR = 4.262, 95% CI: 2.17–8.34), younger group (*P* = .002; OR: 0.973, CI: 0.95–0.99), and alcohol consumption (*P* = .009; OR: 3.368, CI: 1.34–8.42) showed statistical significance. Among 1049 subjects in the nonGBP group, 56 (5.34%) persons were found to have had a GBP at least once in the other series of abdominal sonographies (*P* < .0001).

**Conclusion:**

FLD, older age group, and alcohol consumption are major risk factors of GBP formation in Taiwanese population. The presence of GBPs might be revealed in second-look examinations of abdominal sonographies.

## 1. Introduction

Gallbladder polyps (GBPs) are lesions that project from the gallbladder wall to its lumen.^[1,2]^ GBPs are generally benign lesions; between 3% and 8% of these lesions have the potential to become malignant.^[3–6]^ The reported incidence of GBP ranges from 0.3% to 9.5%, depending on the ethnic demographics and varied dietary habits of the studied populations.^[7–9]^ A study conducted by Cheng-Tang Chiu et al reported a higher prevalence of GBPs in Chinese populations (9.5%) than Western populations (5%).^[9,10]^

Studies have demonstrated associations between GBPs, fatty liver disease (FLD), and metabolic factors.^[7–9,11–15]^ However, controversy about the risk factors for GBP formation, especially in different study populations, remains. These risk factors must be reevaluated.

Abdominal sonography is a convenient and noninvasive modality; that could be used to detect and localize lesions in the abdomen. The detection rate of GBPs has substantially improved since the first application of abdominal sonography,^[2]^ but its interpretation is operator-dependent and thus inconsistent. Screening and prioritized examination programs that target high-risk individuals could be implemented if the risk factors for the development of GBPs were fully identified.

The aim of our study was to identify risk factors for GBPs among Taiwanese populations, whose data were obtained from the General Health Evaluation Center in Taiwan. Additionally, this is the first study to investigate the identification of GBPs from second-look sonography among individuals who underwent abdominal sonography multiple times.

## 2. Material and Methods

### 2.1. Study population

From September 2019 and August 2020, 4862 adults underwent self-paid abdominal sonography for health screening examinations at Chang Gung Memorial Hospital (Kaohsiung, Taiwan). From those 4862 adults, we enrolled 1311 participants through randomization after we excluded subjects with a history of cholecystectomy, hepatectomy, gallbladder stones, polycystic liver disease, malignant liver tumors, and those who experienced only single time of abdominal sonography examination.

**Figure 1.**
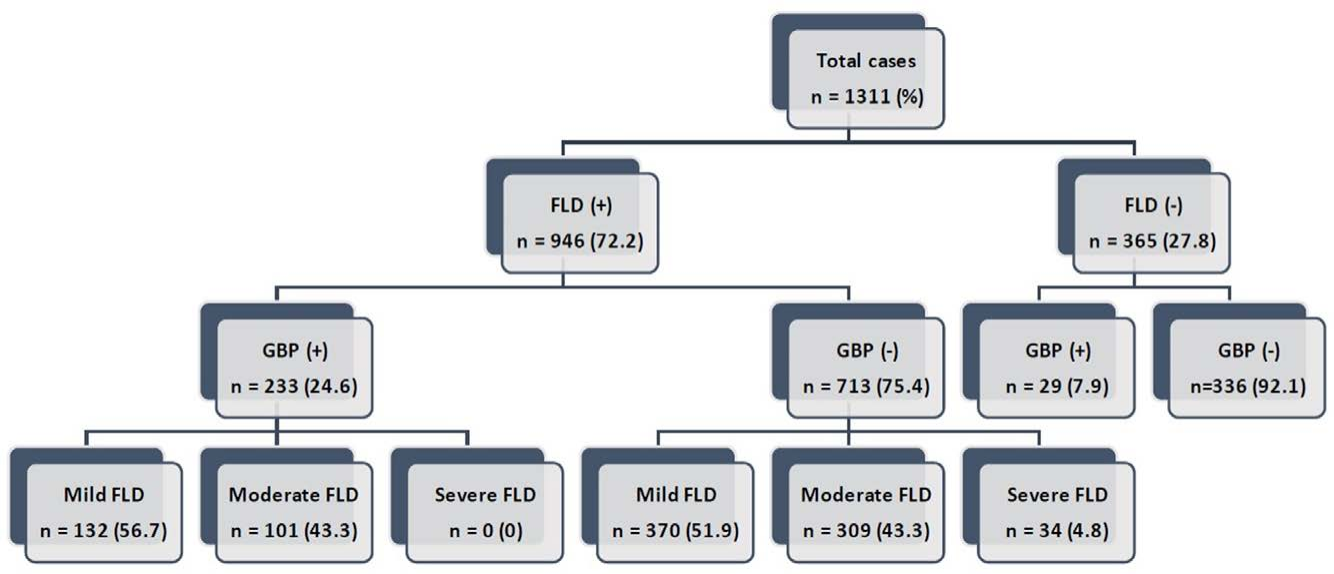
All participants enrolled in this study were divided into subgroups (FLD vs nonFLD).

In Fig 1, all subjects were firstly divided by the existence of FLD, and then, patients were subclassified by the existence of GBP, and finally patients with FLD were further divided according to the severity of FLD. Then, patients were further explored the possibility of the second-look sonographic study for the detection of GBP in FLD.

The study protocol was approved by the Institutional Review Board of Chang Gung Memorial Hospital (IRB No.202001787B0).

### 2.2. Data collection and risk factor analysis

All subjects were divided into 2 groups (a GBP group and a nonGBP group). The study subjects’ baseline characteristics, such as height, age, body weight, body mass index (BMI), sex, waist circumference, blood pressure, history of hypertension, current alcohol consumption, and diabetes mellitus status (whether the subjects had received such a diagnosis) were recorded. Hypertension was determined based on whether a subject’s blood pressure was ≥ 140/90 mm Hg or whether a subject was using an antihypertensive agent. Participants concurrently using glucose-lowering agents (including oral hypoglycemic agents, insulin, and glucagon-like peptide-1 agonists), and participants with a history of having a fasting glucose levels of ≥ 126 mg/dL, a HbA1C level of ≥ 6.5%, or with typical symptoms of hyperglycemia with spot plasma glucose levels of ≥ 200 mg/dL were considered to have diabetes mellitus.^[16]^ Alcohol consumption was defined as the consumption of up to 1 glass of alcoholic beverage per day for women and up to 2 glasses per day for men, in accordance with the Dietary Guidelines for Americans, 9th Edition.^[17]^ Analyses of subjects’ hemograms, serum biochemistry, lipid profiles, and viral hepatitis profiles were conducted. A subject was considered to have hepatitis B virus (HBV) if their sample tested positive for the HBV surface antigen. Similarly, hepatitis C virus (HCV) infection was determined when a subject’s antiHCV test was positive.

### 2.3. Ultrasound

All subjects completed an overnight fast of ≥ 8 hours prior to their health examinations. Specialists in gastroenterology and hepatology conducted abdominal sonographies using a real-time scanner that had a 3.5-MHz array transducer (Toshiba Aplio XG ultrasound system, Zoetermeer, Netherlands) to identify the presence of FLD and GBPs. The detection of GBP was properly and strictly made in all abdominal sonography study.

GBPs were identified as hyperechoic and immobile projections that protruded from the gallbladder wall without casting acoustic shadow; and that occupied fixed positions secondary to shifts in the examinee’s posture.^[1,2,6,18]^ The maximal diameter (GBP < 5mm/ GBP ≥ 5mm) and quantity of GBPs (single/ multiple) were measured during abdominal sonography.

The diagnosis and severity grading of FLD were based on the subjective assessment of liver parenchymal echogenicity, as detailed in other studies.^[19,20]^ The grades of FLD are as follows: mild to slightly increased liver echogenicity, with clear visualization of hepatic and portal vein walls; moderate to diffusely increased liver echogenicity, slightly obscuring the hepatic and portal vein walls; and severe to marked increases in liver echogenicity with deep attenuation that impaired evaluations of the diaphragm and vascular architecture.

“Second-look sonography” was defined as subjects receiving at least 2 times of abdominal sonography on any date in our hospital. We retrospectively reviewed all participants’ every report of abdominal sonography examination (i.e. before or after health examination).

### 2.4. Statistical methods

MedCalc software was used to perform statistical analyses (version: 19.1.5, MedCalc Software Ltd. Acacialaan 22, 8400 Ostend, Belgium). To compare the continuous variables of the groups in the present study, a 1-way analysis of variance and the Mann–Whitney U test were used to make comparisons between groups. Pearson’s chi-squared test and Fisher exact test were used to analyze categorial data. Univariate analysis and multivariate analysis were performed to evaluate the risk factors for subjects with gallbladder polyps. Univariate analysis results are expressed by the odds ratio (OR) and 95% confidence interval (CI). Risk factors associated with GBP were evaluated by stepwise multivariate analysis with logistic regression model. A *P* value of < 0.05 was considered statistically significant.

## 3. Results

### 3.1. Baseline characteristics of the study populations

Of the total 1311 research participants, 262 examinees comprised the GBP group, and 1049 examinees comprised the nonGBP group (Table 1). The overall prevalence of GBP was 19.9% (262/1311). Subjects’ characteristics, metabolic factors, possible risk factors, and alcohol consumption were shown among the GBP group and the nonGBP group (Table 1). GBPs are more commonly found in subjects who aged in fourth and fifth decades (*P* = .0041). The prevalence of GBP was higher in men (77.6% of men vs 22.4% of women *P <* .0001). Body weight, BMI, waist circumference, and alanine aminotransferase (ALT) were significantly higher in the GBP group than in the nonGBP group (*P < .05*). Those in the GBP group had lower high-density lipoprotein (HDL) cholesterol (*P* = .006). Significant differences were found between the GBP group and nonGBP group in terms of the incidence of FLD (88.9% vs 68%, *P* < .0001) and in terms of alcohol consumption (8.8% vs 2.4%, *P* = .0014).

**Table 1:** Clinical profile of all subjects with or without gallbladder polyps.

Variables	GBP (+) (n = 262)	GBP (–) (n = 1049)	*P* value
Age (yr)	48	52	0.0041
Male (%)	203 (77.5%)	569 (54.2%)	<0.0001
Female (%)	59 (22.5%)	480 (45.8%)	
Height (cm)	167.5	167.5	1
Weight (kg)	69.9	66.8	0.0005
Body mass index (kg/m^2^)	24.8	24.4	0.0329
Waist circumference (cm)	84	82	0.0349
Systolic BP (mm Hg)	121	124	0.35
Diastolic BP (mm Hg)	82.5	82	0.89
Hypertension (%)	79 (30.2%)	345 (32.9%)	0.51
Diabetes mellitus (%)	28 (10.7%)	47 (4.5%)	0.48
HBV infection	31 (11.8%)	92 (8.8%)	0.42
HCV infection	2 (0.8%)	33 (3.1%)	0.22
Alpha-fetoprotein (ng*/*ml)	2.49	2.4	0.73
Albumin (g/dL)	4.72	4.67	0.07
AST (IU/L)	22	22	0.73
ALT (IU/L)	24	20	0.045
Fasting glucose (mg/dL)	96	96	0.94
HbA1c (%)	5.6	5.6	0.88
Total cholesterol (mg/dL)	203	200	0.61
Triglyceride (mg/dL)	113	101	0.09
HDL (mg/dL)	45	49	0.006
LDL (mg/dL)	122	119.5	0.1
Fatty liver (by sonography)	233 (88.9%)	713 (68%)	<0.0001
Alcohol consumption	23 (8.8%)	25 (2.4%)	0.0014

Among the 262 subjects in the GBP group, 159 (60.6%) adults were found to have GBPs with a diameter of < 5 mm, and 103 (39.3%) adults were found to have GBPs with a diameter of ≥ 5mm. Most of the examinees (152/262, 58%) had a single GBP during the health checkup.

### 3.2. Analysis of risk factors related to GBPs in subjects with FLD

Among the 1311 subjects, 946 (72.2%) had FLD (Fig. 1). Univariate analysis of metabolic factors among participants with FLD between the GBP group (n = 233) and the nonGBP group (n = 713) indicated that younger age (*P* = .001), male sex (OR = 2.4, CI: 1.74–3.52), greater height (*P* = .006), lower systolic blood pressure (*P* = .027), higher serum albumin levels (*P* = .004), and the presence of hypertension (OR = 2.5, CI: 1.52–4.10) were correlated with GBP formation (Table 2). HCV infection had a strong and negative association with GBP development (OR = 0.04, CI = 0.002–0.66) (Table 2).

**Table 2:** Univariate analysis of metabolic factors associated with fatty liver disease in subjects with or without gallbladder polyps.

	GBP (+)	GBP (-)			95% CI
	(n = 233)	(n = 713)	*P* value	OR	
Age (yr)	48	54	0.0018		
Male (%)	185 (79.4%)	434 (60.9%)	0.0001	2.48	1.74–3.52
Female (%)	48 (20.6%)	279 (39.1%)			
Height (cm)	167.6	165.7	0.0063		
Weight (kg)	71.4	69.1	0.16		
Body mass index (kg/m^2^)	25.3	25.2	0.58		
Waist circumference (cm)	85	85	0.9		
Systolic BP (mm Hg)	121	128	0.0265		
Diastolic BP (mm Hg)	82	83	0.25		
Hypertension (%)	182 (78.1%)	279 (39.1%)	0.0003	2.5	1.52–4.10
Diabetes Mellitus (%)	27 (11.6%)	71 (9.9%)	0.68	1.15	0.59–2.26
HBV infection	27 (11.6%)	68 (9.5%)	0.62	1.19	0.60–2.34
HCV infection	0 (0%)	68 (9.5%)	0.024	0.04	0.002–0.66
Alpha-Fetoprotein (ng/ml)	2.5	2.4	0.94		
Albumin (g/dL)	4.74	4.7	0.0039		
AST (IU/L)	22	23	0.4		
ALT (IU/L)	25	23	0.65		
Fasting glucose (mg/dL)	97	99	0.12		
HbA1c (%)	5.6	5.65	0.26		
Total cholesterol (mg/dL)	205	201	0.9		
Triglyceride (mg/dL)	114	116	0.5		
HDL (mg/dL)	44	46	0.26		
LDL (mg/dL)	124	122	0.39		

Table 3 revealed univariate analysis and multivariate analysis of risk factors for subjects with gallbladder polyps. In a multi-variate analysis for risk factors for GBP formation, FLD (*P* < .0001, OR = 4.262, 95% CI: 2.17–8.34), younger group (*P* = .002; OR: 0.973, CI: 0.95–0.99), and alcohol consumption (*P* = .009; OR: 3.368, CI: 1.34–8.42) showed statistical significance.

**Table 3:** Univariate analysis and multivariate analysis of risk factors for subjects with gallbladder polyps.

	Univariate analysis		Multivariate analysis	
Variables	OR (95% CI)	*P* value	OR (95% CI)	*P* value
Younger age	0.55 (0.33–0.92)	0.023	0.97 (0.96–0.99)	0.0028
Hypertension	0.87 (0.57–1.32)	0.51		
Diabetes mellitus	1.25 (0.84–3.03)	0.48		
HBV infection	1.29 (0.69–2.40)	0.42		
HCV infection	0.23 (0.03–1.75)	0.22		
Fatty liver disease	4.16 (10.67–35.55)	<0.0001	4.26 (2.18–8.35)	<0.0001
Alcohol consumption	3.94 (1.70–9.16)	0.0014	3.37 (1.35–8.42)	0.0094

A strong association between FLD incidence and GBP formation was observed (*P <* .001) (Table 4); however, the severity of FLD did not have a significant relationship with GBP formation (*P* = .052) (Table 5).

**Table 4:** The relationship between fatty liver disease and gallbladder polyps.

	FLD, No (%)	nonFLD, No (%)
GBP (+)	233 (88.9)	29 (11.1)
GBP (–)	713 (68)	336 (32)

**Table 5:** The relationship between the severity of fatty liver disease and the incidence of gallbladder polyps.

Fatty liver disease*	Mild, No (%)	Moderate, No (%)	Severe, No (%)
GBP (+)	132 (50.3)	101 (38.5)	0 (0)
GBP (–)	370 (35.2)	309 (29.5)	34 (3.2)

### 3.3. GBPs in second-look of abdominal sonography

**Figure 2.**
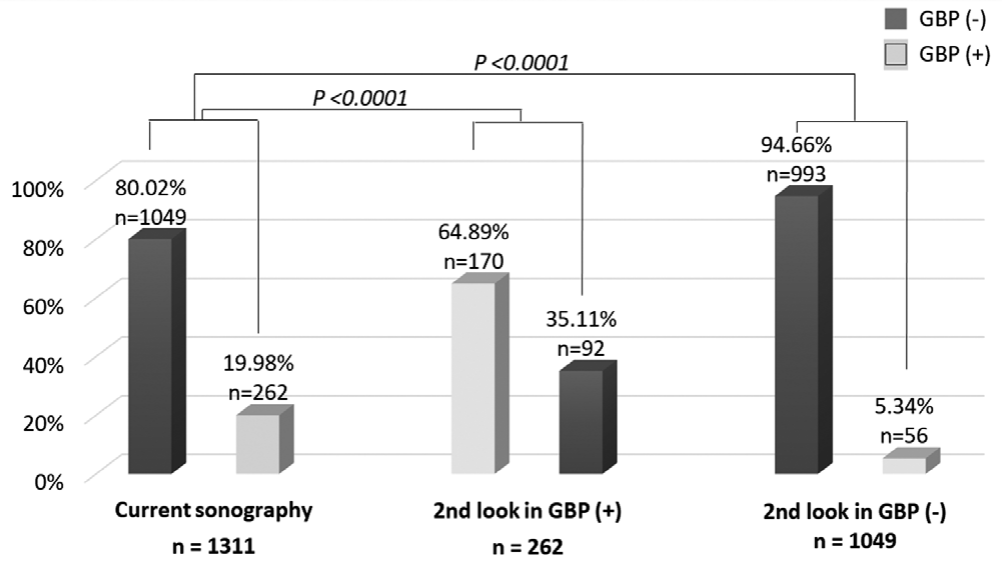
Second-look sonographic identification of gallbladder polyps in all subjects.

As demonstrated in Figure 2, GBPs were detected in (262/1311, 19.98%) subjects (GBP group), and (1049/1311, 80.02%) participants were found to have no GBPs (nonGBP group). During second-look sonography study in the GBP group subjects (n = 262), GBPs were ever missed at least once in another abdominal sonography series (n = 92/262, 35.1%), *P* < .0001; additionally, during second-look sonography study in the nonGBP group (n = 1049), there were 56/1049 (5.34%) found to have GBPs at least once in another series of abdominal sonography examinations (*P* < .0001).

## 4. Discussion

The identification of GBPs has increased as a result of the increasingly universal use of high-resolution abdominal sonography during regular checkup.^[8,9,15,21]^ Due to dietary changes (such as high-calorie and high-fat diets), the prevalence of metabolic syndrome has increased, and it had even been reported to be one of the risk factors for GBP formation.^[7,8,11,12,14,15]^ This might also contribute to the increasing prevalence of GBP. Our study demonstrated a significantly higher prevalence of GBP (19.9%) and higher proportion of fatty liver disease (FLD) in the GBP group (90%) than those reported in other studies.^[7–9,14]^

The association between hepatic fat and GBP formation was reported by Lim et al in 2015^[14]^ based on a cohort of 2643 Korean individuals who attended screening health examinations during the study period. Lim et al postulated that hepatic fat, which is anatomically close to GB fossa, may play a more dominant role than visceral adipose tissue plays in the development of GBPs. We found the incidence of GBP to be associated with the prevalence of FLD, but GBP incidence had no association with FLD severity.

The present study clarifies that men in an ethnically Taiwanese populations had a higher prevalence of GBP development than women in the same populations. Additionally, the mean age in the GBP group was below that in the nonGBP group. As reported in the study conducted by Cheng-Tang Chiu et al, GBPs were more common in middle-aged patients, peaking at age 41–50 years.^[9]^

Alcohol exposure was also considered as risk factors for GBPs in our study. This finding was inconsistent with other researches. An animal experimental study and population-based study revealed that alcohol consumption had a protective effect against GBP formation because it results in reduced biliary cholesterol saturation.^[22,23]^ The inconsistent results may be due to the various study designs and different volume of alcohol in beverages. Further studies are necessary to determine the mechanism and roles of alcohol consumption in the development of GBPs.

In accordance with National Cholesterol Education Program (NCEP) Adult Treatment Panel (ATP) III,^[24]^ the present study determined metabolic syndrome, to be present when 3 or more of the following measurements were noted: abdominal obesity (waist circumference of > 101.6 cm in men, and > 88.9 cm in women); triglyceride (TG) levels of 150 mg/dL, HDL cholesterol levels of > 40 mg/dL in men or > 50 mg/dL in women; systolic blood pressure of ≥ 130 mm Hg, or diastolic blood pressure of ≥ 85 mm Hg; or fasting glucose levels of ≥ 100 mg/dL.

The relationship between GBP and metabolic syndrome has been well-documented in the literature.^[12,15]^ Our study provided a perspective on a cohort from Taiwan, a country in east-Asia, which has higher prevalence of viral hepatitis yet a lower prevalence of obesity than Western countries.^[25]^ A correlation between metabolic syndrome and GBP was also observed in our study. We found that those in the GBP group had significantly higher body weight, higher BMI, larger waist circumference, lower HDL cholesterol, and a higher prevalence of hypertension those in the nonGBP group (*P* < .05). Multivariate analysis revealed no statistical significance between the 2 groups in terms of these metabolic factors.

Many factors affect the incidence of GBP formation, and the most common type of GBPs are cholesterol polyps.^[6]^ Salmenkivi et al hypothesized that GBPs are formed when direct cholesterol is deposited in the gallbladder in a process, similar to that of the formation of plaque in arteriosclerosis.^[26]^ Tilvis et al reported that free cholesterol can transform from bile to gallbladder mucosa, and intrahepatic cholesterol synthesis changes are related to the development of cholesterol polyps.^[27]^ In the present study, we did not reveal that plasma cholesterol, LDL, or TG levels were higher in the GBP group than in the nonGBP group. Currently, the association between plasma T-Cho/TG/LDL levels and the formation of polyps in the gallbladder remains controversial.^[28–31]^ Further investigation is required to clarify whether the cholesterol deposited originates in plasma.

The mechanism behind the relationship between hypertension and GBP required clarification. However, the incidence of metabolic syndrome and obesity correlated with the incidence hypertension, which might explain the association between the incidence of hypertension and the development of GBP.^[25]^

HBV infection is endemic in Taiwanese populations, and might directly affect abnormal changes of the gallbladder, such as thickening of the gallbladder wall and alterations to gallbladder volume and contents.^[32]^ Chronic hepatitis B has been reported to be a risk factor for GBPs in several studies^[7–9,11,12]^; however, a few studies have reached the opposite conclusion.^[33,34]^ Our study did not identify a positive association between GBPs and chronic hepatitis B. Furthermore, statistical results suggested that GBPs have a negative association with the incidence of HCV infection in subjects with FLD, which had never been reported in the literatures. The inconsistent findings may be due to the number of subjects, the ethnicity of the patients, and other factors such as the strategy of antiviral therapy. The pathophysiological relationship between GBPs and viral hepatitis remains unknown; thus, further study is required to clarify the relationship.

To our knowledge, this is the first study to evaluate the second-look abdominal sonography reports in detecting GBPs. In at least one abdominal sonographic study, 5.3% of the subjects in the nonGBP group found GBPs after further exams; furthermore, 35.1% of participants of the GBP group were found to have missed GBPs in at least 1 abdominal sonography examination. In our current study, the reports of first-look abdominal sonography study for the detection of GBP were considered to be reliable. In fact, series of abdominal sonographies were properly and strictly performed. There still existed some inconsistency results for GBP detection in the same individuals (Fig. 2). Three factors might be behind this phenomenon, which will be emphasized in this study: (1) the operator’s technical competence and subjective interpretation of the abdominal sonography, (2) fluctuation in GBP size and the presence or extent of gallbladder contraction at each abdominal sonography study, and (3) the extension of subjects’ abdomen during examination.

The present study had some strengths. First, all healthy participants, which is representative to generalized population underwent comprehensive measurements and tests, (hemograms, serum biochemistry, lipid profiles, and viral hepatitis profiles), available for the present study’s investigation of the factors behind GBP formation. Second, this is the first study to investigate the second-look GBPs in the same individuals underwent abdominal sonography multiple times; the fact that this is the first such study emphasizes that conducting such a study requires high-quality technology and the intent to determine the presence of GBPs during operator-dependent sonography.

Out study had several limitations. First, this was a retrospective study of data from a single medical center, and selection bias may have arisen. Second, the specific amount of daily alcohol consumption could not be recorded, and questionnaire such as the Alcohol Use Disorders Inventory Test (AUDIT) was not used^[35]^ to define alcohol use disorder. Third, the histopathologic type of GBP could not be determined through abdominal sonography in health examinations. Fourth, the sonographic grading of FLD only acts as a semiquantitative grading tool that was unable to accurately reflect the severity of FLD. The subjective interpretation and differences in technique between sonographic operators might also vary the rate of GBP diagnosis. Finally, the interval between the first and the second abdominal sonography exam was not constant in every subjects. When intervals between the first and second sonography exam were too long, there might be the possibility that sonographer detected a newly developed gallbladder polyp. Further high-quality and prospective studies may be necessary.

## 5. Conclusions

In conclusion, FLD, older age group, and alcohol consumption are major risk factors in Taiwanese populations for the formation of GBPs. The severity of FLD bears no correlation with the incidence of GBPs. In the FLD subgroup, HCV infection was found to be negatively associated with GBP incidence. Furthermore, examiners should be more cautious about performing operator-dependent abdominal sonography on patients who are at a high risk of developing GBPs. GBPs might be identified in second-look abdominal sonography.

## Author contributions

Study concept and design: KWC and SHL; data collection: SHL and YCC; data analysis and interpretation: SHL, and KTW; and manuscript drafting and critical revisions: SHL, KWC, and CCW.

## Footnotes

* Through sonography.
